# Infective endocarditis caused by Salmonella enteritidis in a dialysis patient: a case report and literature review

**DOI:** 10.1186/1471-2334-9-161

**Published:** 2009-09-29

**Authors:** Yusuke Tsugawa, Miyuki Futatsuyama, Keiichi Furukawa, Fumika Taki, Yuji Nishizaki, Keiichi Tamagaki, Yuki Kaneshiro, Yasuhiro Komatsu

**Affiliations:** 1 Department of Nephrology, Division of Internal Medicine, St. Luke's International Hospital, Tokyo, Japan; 2 Department of Infectious Disease, Division of Internal Medicine, St. Luke's International Hospital, Tokyo, Japan

## Abstract

**Background:**

Infective endocarditis is significantly more common in haemodialysis patients as compared with the general population, the causative pathogen is generally *Staphylococcus aureus; *there have been no previously reported cases of infective endocarditis caused by a *Salmonella *species in haemodialysis patients.

**Case Presentation:**

We report the case of a 68 year-old woman on haemodialysis who developed infective endocarditis as a result of Salmonella enteritidis. Although we treated the patient with ceftriaxone combined with ciprofloxacin, infective endocarditis was not detected early enough and unfortunately developed into cerebral septic emboli, which ultimately resulted in death.

**Conclusion:**

Although there are several reports that Salmonella endocarditis without cardiac failure can be successfully treated with antibiotics alone, early surgical intervention is essential for some cases to prevent life-threatening complications. Transesophageal echocardiography should be performed in any patient with high clinical suspicion of infective endocarditis. To the best of our knowledge, this is the first case-report of Salmonella endocarditis in a haemodialysis patient.

## Background

Infective endocarditis is significantly more common in haemodialysis patients as compared with the general population. In addition to the four conventional categories of infective endocarditis (native valve infective endocarditis, prosthetic valve infective endocarditis, infective endocarditis in intravenous drug users, and nosocomial infective endocarditis), a fifth category, health-care associated infective endocarditis or haemodialysis associated infective endocarditis, has recently been proposed. Although infective endocarditis is a common complication among haemodialysis patients, the causative pathogen is generally *Staphylococcus aureus; *there have been no reported cases of infective endocarditis caused by a *Salmonella *species in haemodialysis patients. *Salmonella *species have been reported to cause bacteremia [1] and infective endocarditis in 5 and 1.4% of patients [2] during gastrointestinal infections, respectively. However, in general, cases of infective endocarditis due to *Salmonella *species are relatively rare. Here, we report the case of a 68 year-old woman on haemodialysis who developed infective endocarditis on her native mitral valve as a result of Salmonella enteritidis. After hospitalization, the patient was treated with intensive antibiotic therapy. However, the infective endocarditis was not detected early enough and unfortunately developed into cerebral septic emboli, which ultimately resulted in death.

## Case Presentation

A 68 year-old Asian woman was admitted to our hospital presenting with a two-day history of high grade fever, diarrhea and nausea. The patient's symptoms began one day after eating grilled chicken and beef at a restaurant; however, she did not complain of abdominal pain, vomiting, or hematochezia. The patient was diagnosed with diabetes mellitus at the age of 50, had undergone haemodialysis due to diabetic nephropathy for the past 2 years, and was under three times weekly haemodialysis. She had also undergone an aortic mechanical valve replacement operation for aortic stenosis 7 months prior to the current admission.

Upon admission to our hospital, the patient's height was 149 cm and her body weight were 61.0 kg (dry weight). The patient's temperature was 106°F, blood pressure was 150/90 mmHg, heart rate was 152/min, and respiratory rate was 24/min. Cardiovascular examination revealed a Levine IV/VI systolic murmur at the right second intercostal space. Her bowel movements were slightly hypoactive and physical examination did not reveal any abdominal tenderness or rebound. No evidence of embolization including Osler node, Janeway lesion and petechiae was observed. No other abnormalities were noted on the systemic examination. Initial laboratory examinations revealed the following results: hemoglobin, 115 g/L [110-146 g/L]; total leukocyte counts, 10300/μL [3500-8200/μL] without left shift (Myelo 0.5%, Meta 0.5%, Stab 2.0%, Seg 90.0%, Lym 1.5%, Mono 5.5%); normal platelet counts, 1.42 × 10^3^/μL [1.40-3.89 × 10^3^/μL]; significantly elevated C-reactive protein, 18.54 mg/dL [<0.30 mg/dL]; Na 131 mEq/L, K 4.3 mEq/L, Cl 97 mEq/L, HCO3- 18.9 mmol/L; BUN 43.6 mg/dL, Cre 7.25 mg/dL (before haemodialysis); serum glucose, 173 mg/dL; and HbA1c, 7.3% [4.3-5.8%]. In addition, the patient was tested negative for HIV antibodies.

**Figure 1:**
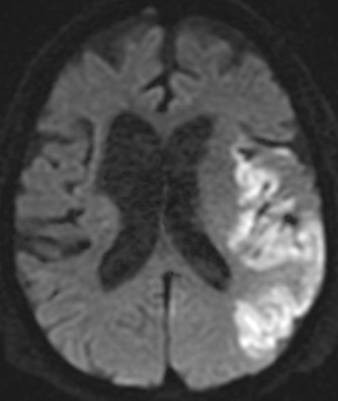
**Diffusion-weighted image of the brain MRI taken on day 9 with high intensity areas on the left middle cerebral artery region**.

**Figure 2:**
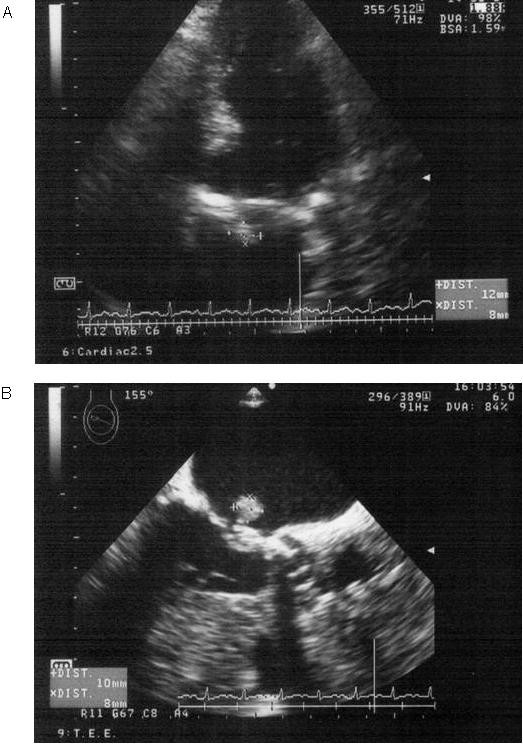
**Transthoracic echocardiogram (A) and transesophageal echocardiogram (B) performed on day 13 revealed vegetation on the mitral valve**.

**Figure 3:**
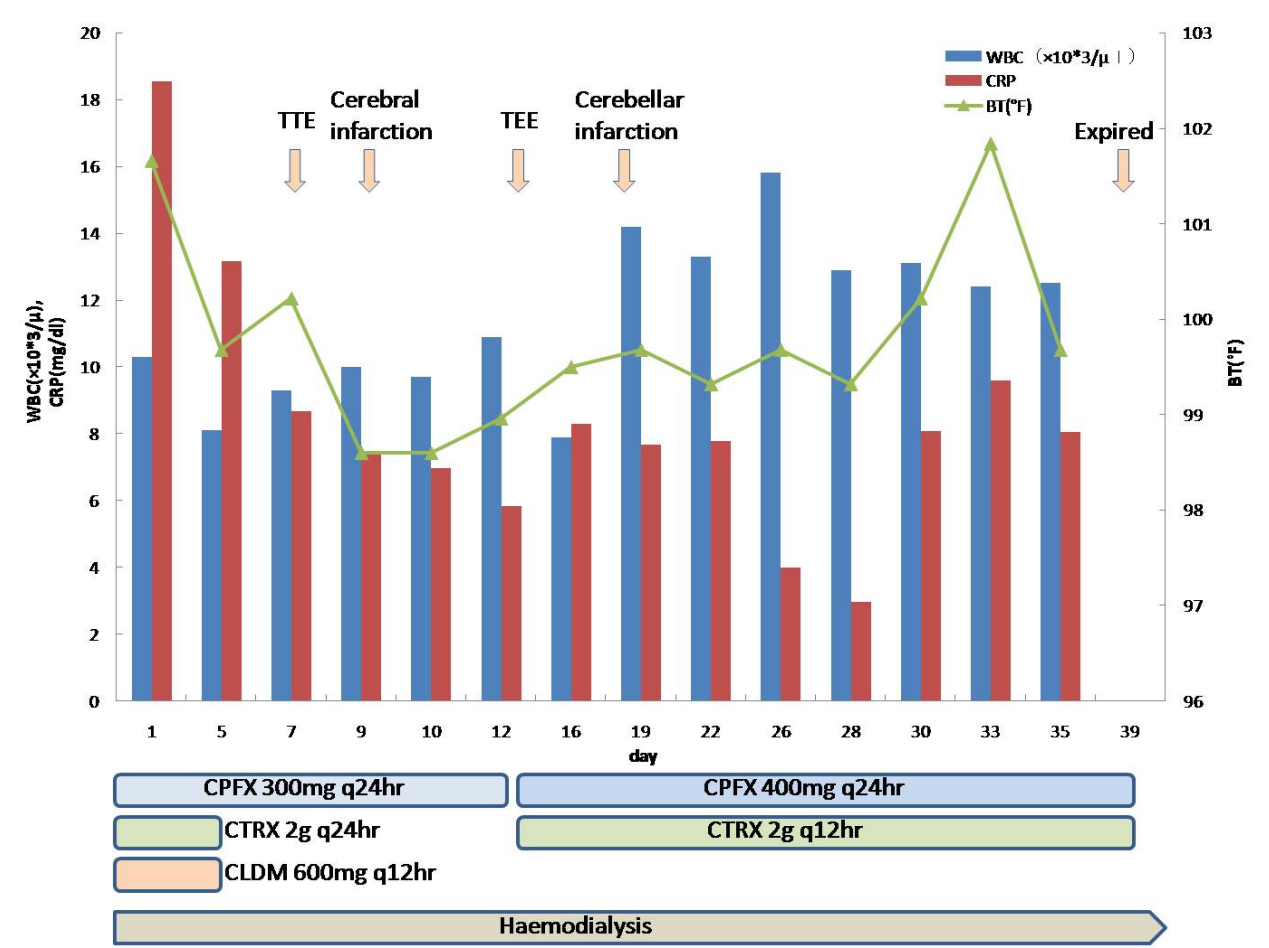
**Laboratory data, body temperature, and antibiotics administered to the patient after admission to the hospital**. Abbreviations: WBC; white blood cell, BT; body temperature, CRP; C-reactive protein, TTE; transthoracic echocardiography, TEE; transesophageal echocardiography, CPFX; ciprofloxacin, CTRX; ceftriaxone, CLDM; clindamycin

**Figure 4:**
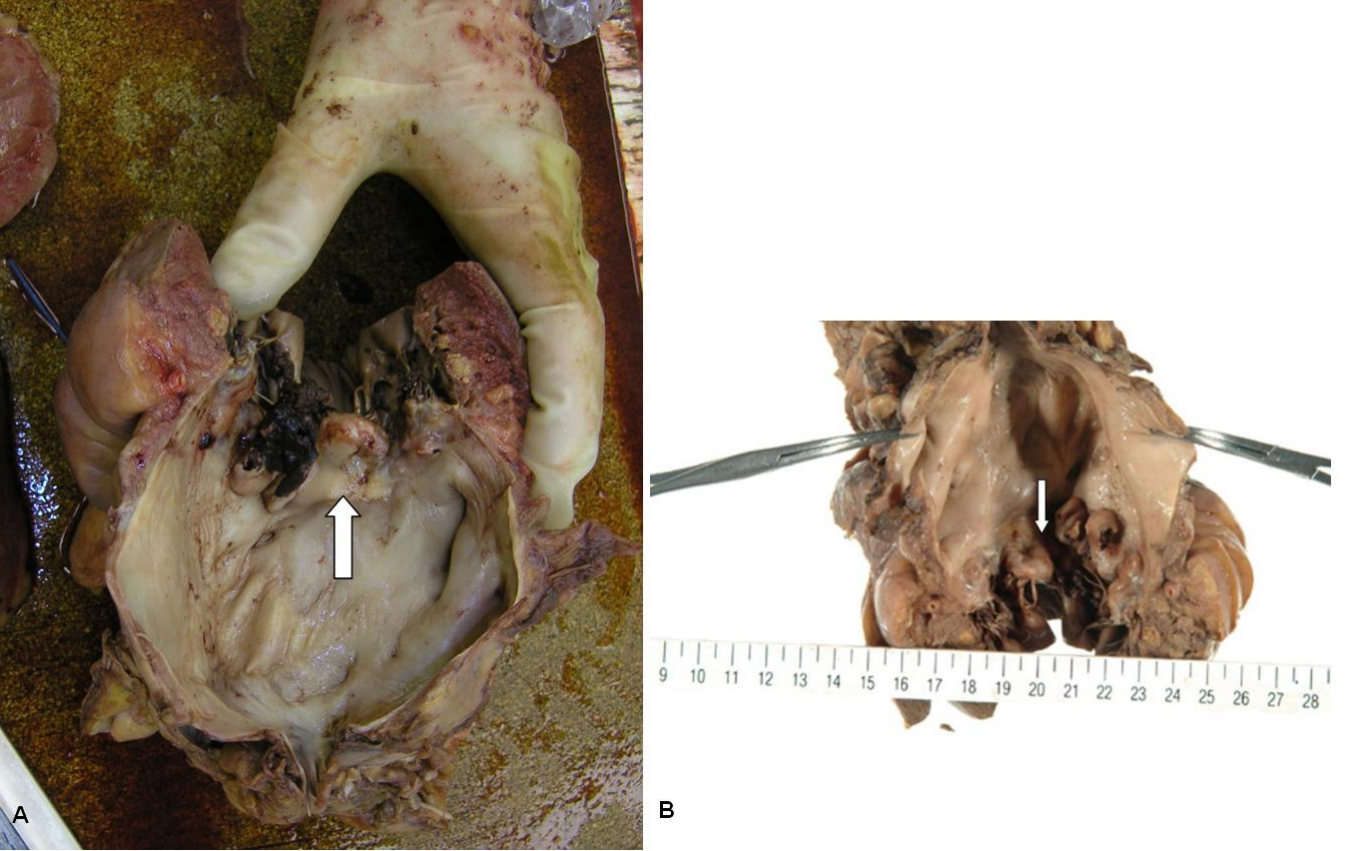
**A rigid node on the mitral valve (arrow), found during autopsy (A, B)**.

**Figure 5:**
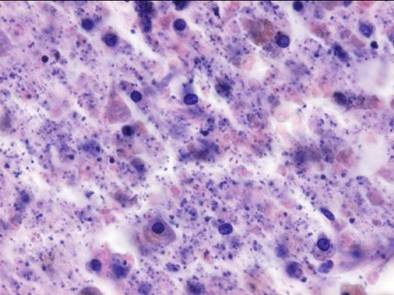
**Bacterial proliferation in the cerebral infarction**.

Salmonella enteritis or campylobacter enteritis was suspected due to the history of eating grilled chicken. Treatment was initiated with ciprofloxacin 300 mg q24hr, ceftriaxone 2 g q24hr, and clindamycin 600 mg q12hr intravenously, started on day of admission. Three days post-admission, a serial blood culture taken on admission revealed Salmonella enterica serotype Enteritidis (O9, H-g) that was sensitive to ceftriaxone (MIC <= 1) and ciprofloxacin (MIC <= 0.25) (Table 1). After detection of the causable microorganism, ceftriaxone and clindamycin treatment was ceased, and the patient was treated with ciprofloxacin alone. Since the patient's aortic valve was a prosthetic, and *Salmonella *species have a high affinity for intravascular intima, there was a concern about prosthetic infective endocarditis. Therefore, the patient underwent transthoracic echocardiography (TTE) on day 7; however, no abnormalities were detected by TTE. On day 9, the patient showed a sudden disturbance in mental status, left conjugate deviation, and right hemiplegia. Diffusion-weighted imaging of the brain via MRI showed an acute cerebral infarction of the middle cerebral artery region (figure 1). Following this discovery, the TTE was re-examined, followed by a transesophageal echocardiography (TEE) on day 13. The TEE revealed floppy vegetation on the mitral valve, which was 12 × 8 mm in size (figure 2). We could not find any aberrance of the replaced aortic valve. Although surgery to remove the vegetation was considered, acute cerebral infarction contraindicated her from undergoing the operation. The ciprofloxacin dose was increased to 400 mg q24hr and was combined with ceftriaxone 2q q12hr, on day 12. Consequently, the patient was treated solely with ciprofloxacin for 8-day period (from day 4 to day 11). Although the patient's neurological deficit ameliorated gradually, she experienced another infarction at the left occipital lobe and the right cerebellar hemisphere on day 18. Conservative therapy with antibiotics was continued; however, her mental status progressively decreased and blood pressure deteriorated. Blood culture examinations were repeated, but they were all negative. haemodialysis ceased on day 35 due to the unstable haemodynamic status, although treatment with ciprofloxacin and ceftriaxone were continued. On day 39, the patient died of respiratory failure and cardiac arrest. Her clinical course is shown in figure 3. Autopsy revealed a rigid node on the mitral valve (figure 4), associated with septic emboli (mass of gram-negative microorganisms) at the left occipital lobe (figure 5).

**Table 1:** Susceptibility of the Salmonella Enteritidis to the Antibiotics

Antibiotics	MIC	Sensitivity
CEZ	<=4	R
AMK	<=2	R
TOB	<=1	R
GM	<=1	R
AZT	<=1	S
CTRX	<=1	S
CAZ	<=1	S
ABPC	<=2	S
PIPC	<=4	S
CPFX	<=0.25	S
ST	<=20	S
S/A	<=2	S
MEPM	<=0.25	S
CFPM	<=1	S
PIPC/TAZ	<=4	S

Abbreviations: CEZ; cefazolin, AMK; amikacin, TOB; tobramycin, GM; gentamicin, AZT; aztronam, CTRX; ceftriaxone, CAZ; ceftazidime, ABPC; ampicillin, PIPC; piperacillin, CPFX; ciprofloxacin, ST; sulfamethoxazole-trimethoprim, S/A; ampicillin sulbactam, MEPM; meropenem, CFPM; cefepime, PIPC/TAZ; piperacillin-tazobactam

## Discussion

Infective endocarditis is a common complication of haemodialysis, the incidence of which is 1.7-2.0 cases/1000 patients in France (50-60 times higher than the general population), and 483 cases per 100,000 person-years in the United States of America (74 times higher than the general population) [3,4]. The most common causable pathogen is *Staphylococcus aureus*, which contributes to 40-80% of the cases (Table 2). A report from Duke University Medical Center during 1993-1999 demonstrated that the overall proportion of haemodialysis patients out of a total of 329 infective endocarditis patients increased from 6.7% to 20% over the 7 year study period. In addition, there was a significant increase in *Staphylococcus aureus *as the causable agent [5]. We experienced 46 infective endocarditis patients in our hospital during 2003-2008, 4 of which were haemodialysis patients (8.7%) [6]. The mitral valve (up to 50% of cases) and the aortic valve (up to 40% of cases) are the most commonly affected valves for infective endocarditis in haemodialysis patients [7-9]. To the best of our knowledge, this is the first English report of Salmonella infective endocarditis in a haemodialysis patient.

**Table 2:** Previously published reports of causable organisms for infective endocarditis in haemodialysis patients

	Nori et al. [17]	Spies et al. [9]	Doulton et al. [18]	Maraj et al. [8]	McCarthy & Steckelberg [7]
Episodes of infective endocarditis	N = 54	N = 40	N = 30	N = 30	N = 17
Staphylococcus aureus	40%	50%	63%	80%	40%
MRSA	20%	15%	7%	23%	
CNS	22%	12%	13%	3%	10%
Enterococcus sp.	33%	23%	10%	7%	20%
Streptococcus sp.			10%	3%	25%
**Gram-negative sp**.	**13%**	**10%**			
Candida sp.		3%		3%	
Aspergillus sp.					5%

Negative blood culture	2%	10%			

Gastroenteritis due to *Salmonella *species occurs relatively frequently; however, it is a rare cause of infective endocarditis. Guerrero identified a total of 72 cases of Salmonella endocarditis within the English literature in 2004; 42 cases before 1987 and 30 cases in 1987-2004 [10]. In a population-based study, Nielsen reported 111 cases of Salmonella bacteremia from 1994 through 2003 in North Jutland Country, Denmark. Among them, there were only 2 cases (1.8%) of endocarditis documented [11]. Transient bacteremia is reported to be present in 5-10% of patients with Salmonella gastroenteritis. Within these cases, the incidence of infective endocarditis is approximately 2.0% [11].

The intravascular intima is vulnerable to *Salmonella *species, thus reflecting the ability of salmonella to cause endothelial infections in the presence of atherosclerosis. The risk of endovascular focal infection in elderly patients (age>50) with Salmonella bacteremia is reported to be 7-23% [10-12]. The prognosis of Salmonella endocarditis is improving; Cohen reported the mortality of 69% (29/42) in 1987 [13], where as Guerrero identified the mortality of 20% (6/30) in 1987-2004 [10]. Guerrero concluded that surgery has played an essential role in reducing the mortality of Salmonella endocarditis, and surgical intervention, including valve replacement, increases survival in Salmonella endocarditis and is the treatment of choice for patients with cardiac failure, persisting sepsis and for those who relapse after discontinuation of antimicrobial therapy. Early surgical intervention may prevent life-threatening complications including cerebral infarction [14,15], as was observed in the current case. In this case, *Salmonella *species were suspected from the medical history. Therefore, we began the appropriate treatment of ceftriaxone combined with ciprofloxacin, and performed an early investigation with TTE; however, we failed to detect infective endocarditis before it induced cerebral infarction. The cerebral infarction contraindicated her from early surgical intervention. Owing to the increased sensitivity of TEE over TTE in detecting vegetations (Sensitivity for native valve: TTE 60-65%, TEE 85-95%) [16], TEE should be performed in any haemodialysis patient with high clinical suspicion of infective endocarditis, and whenever bacteremia with organisms typical for infective endocarditis was detected. It should also be emphasized that in patients with prosthetic valve, TEE is mandatory for those suspected to have infective endocarditis, owing to unacceptably low sensitivity of TTE for detecting infective endocarditis of the prosthetic valve (Sensitivity for prosthetic valve: TTE <50%, TEE 82-90%) [16]. Identification of the optimal treatment for Salmonella endocarditis requires further investigation. In addition, the emergence of ceftriaxone-resistant *Salmonella *species has recently been reported [17,18]. Due to this resistance, combination treatment with a third generation cephalosporin and a fluoroquinolone is offered in life-threatening infections. We treated the patient in this case study with ciprofloxacin, and later combined with ceftriaxone; however, we failed to prevent the patient from developing cerebral infarction, which was identified as septic emboli upon autopsy.

## Conclusion

Salmonella endocarditis has been previously reported in the general population as a rare complication of Salmonella gastroenteritis. To the best of our knowledge, this is the first case-report of Salmonella endocarditis in a haemodialysis patient. Salmonella endocarditis may present an aggressive course; therefore, early surgical intervention is essential for some cases to prevent life-threatening complications. Non-surgical management may treat the patient successfully, but further investigation is required to distinguish patients who require an expeditious surgical intervention from those who can be treated with antibiotics alone.

## Competing interests

The authors declare that they have no competing interests.

## Authors' contributions

YT, MF, KF and YK designed the concepts and design. FT, YN, KT and YK collected the data and contributed to the critical revision of the paper. All authors read and approved the final manuscript.

## Pre-publication history

The pre-publication history for this paper can be accessed here:

http://www.biomedcentral.com/1471-2334/9/161/prepub
